# Unveiling early stage autoimmune gastritis: novel endoscopic insights from two case reports

**DOI:** 10.3389/fimmu.2024.1416292

**Published:** 2024-06-17

**Authors:** Yunfeng Yu, Xueli Shangguan, Rong Yu, Yangpeng Wu, En Xu, Chuanchuan Tan

**Affiliations:** ^1^ Digestive Endoscopy Center, The First Hospital of Hunan University of Chinese Medicine, Changsha, Hunan, China; ^2^ School of Traditional Chinese Medicine, Hunan University of Chinese Medicine, Changsha, Huanan, China

**Keywords:** autoimmune gastritis, early-stage, endoscopy, collecting venules, yellowish-white cobblestone-like elevations

## Abstract

The predominant characteristic of autoimmune gastritis (AIG) is corpus-dominant advanced atrophy, which is mostly observed in the middle to late stages. More reports are needed on the endoscopic features of the early stage. In this report, we present two cases of early-stage AIG in which endoscopic examinations showed no atrophy of the gastric mucosa but displayed a transition of collecting venules from a regular to an irregular arrangement. In addition, yellowish-white cobblestone-like elevations were observed in the fundic gland region. Histologically, the observed manifestations included pseudohypertrophy and protrusion of parietal cells into the lumen, possibly along with hyperplasia of G cells, lymphocytic infiltration and potentially pseudopyloric gland metaplasia. Serologically, the anti-parietal cell antibody returned positive results, whereas the anti-intrinsic factor antibody yielded negative results. In this study, we summarized some endoscopic features of two patients, aiming to provide clues for endoscopists to detect early-stage AIG.

## 1 Introduction

Autoimmune gastritis (AIG) is a type of atrophic gastritis associated with autoimmunity (1, 2) and is associated with an increased risk of gastric neuroendocrine tumors and pernicious anemia (3–5). Early identification and management of AIG are particularly important ways to improve patient prognosis. Nonetheless, patients with AIG often exhibit atypical clinical symptoms (6, 7), necessitating a reliance on endoscopic imaging, serological markers, and other diagnostic tools for accurate identification (8–10). Among these, endoscopic imaging is the most critical opportunity for detection, determining the necessity for further biopsy and serving as an important criterion for diagnosing AIG (11, 12). The characteristic endoscopic manifestation of AIG is the homogeneous atrophy of the gastric body with antral sparing. Simultaneously, other features include remnant oxyntic mucosa, sticky adherent dense mucus, and hyperplastic polyps (13). These characteristic endoscopic manifestations play a key role in diagnosing AIG, but are more commonly observed in the middle to late stages than in the early stage (14). This challenge complicates the endoscopists’ ability to identify early-stage AIG during diagnosis, leading to missed opportunities for comprehensive evaluation and a low rate of confirmed early-stage AIG diagnoses.

This study reported two cases of suspected early-stage AIG based on endoscopic manifestations that were confirmed by further serological and histopathological examinations. In this study, we summarize the endoscopic characteristics observed in early-stage AIG cases: non-atrophic gastric mucosa, irregular arrangement of localized collecting venules (CVs) in the fundic gland region, yellowish-white cobblestone-like elevations, and mild gastric mucosal swelling or non-swelling. We aimed to provide insights for enhancing the endoscopic diagnosis of early-stage AIG, thereby increasing the rate of clinical confirmation.

## 2 Case 1

A 41-year-old Chinese man underwent an upper gastrointestinal endoscopy because of a burning sensation in the upper abdomen. He had never undergone eradication therapy for *Helicobacter pylori* (*H. pylori*) infection and was not taking any medications, including proton pump inhibitors or antibiotics. Both his grandfather and uncle had rheumatoid arthritis.

**Figure 1:**
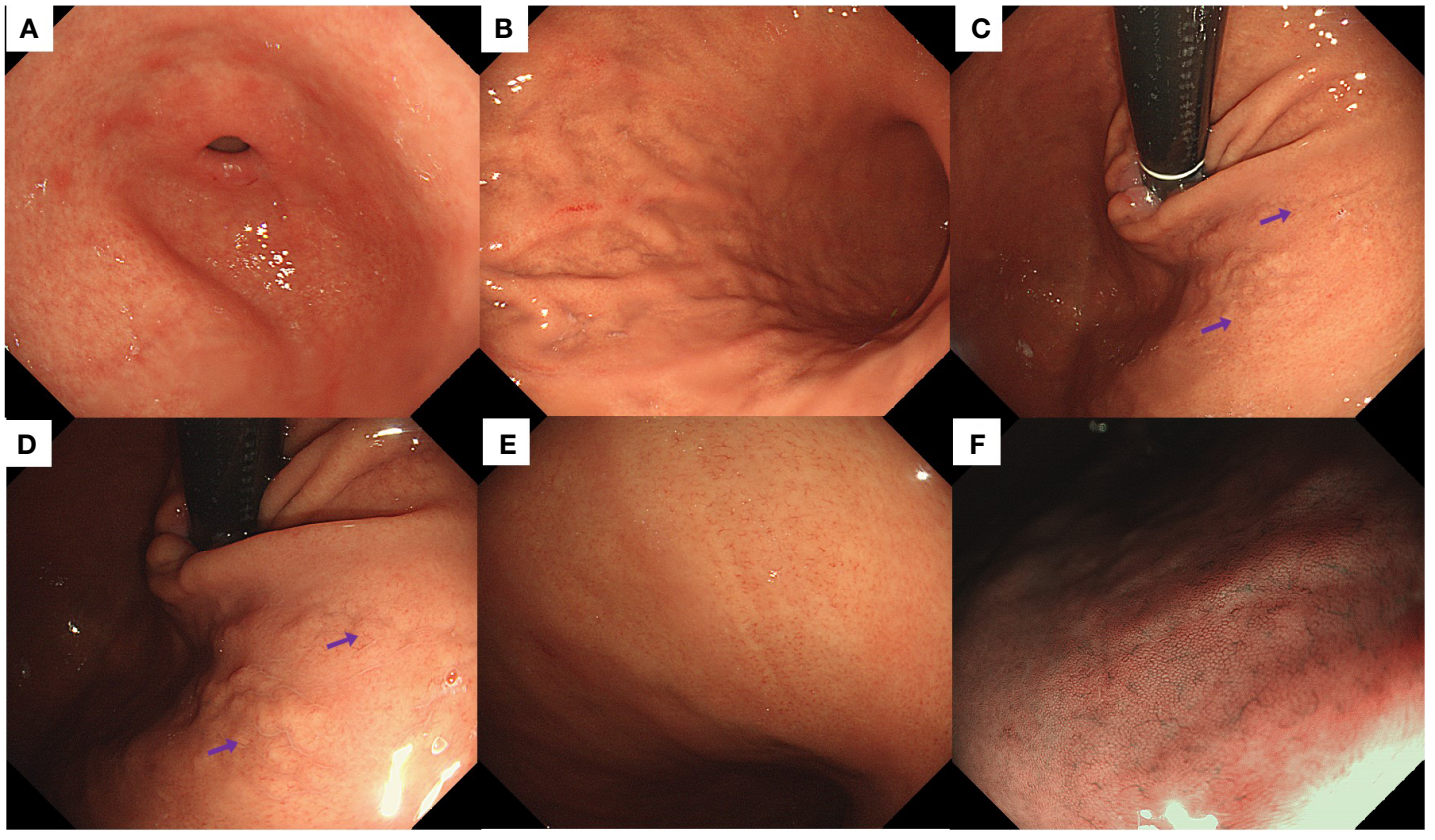
Endoscopic findings of the patient in Case 1. **(A)** Normal antrum. **(B)** The greater curvature of the corpus displayed an irregular distribution of folds, with deep fissure-like structures and localized yellowish-white discoloration. **(C, D)** Yellowish-white cobblestone-like elevations were noted in the lesser curvature of the corpus, accompanied by irregular distribution of CVs (purple arrows) on the surface. **(E)** Whitish areas with elongated and widened CVs were detected in the lesser curvature of the corpus. **(F)** Magnifying endoscopy with narrow-band imaging did not reveal significant mucosal swelling.

**Figure 2:**
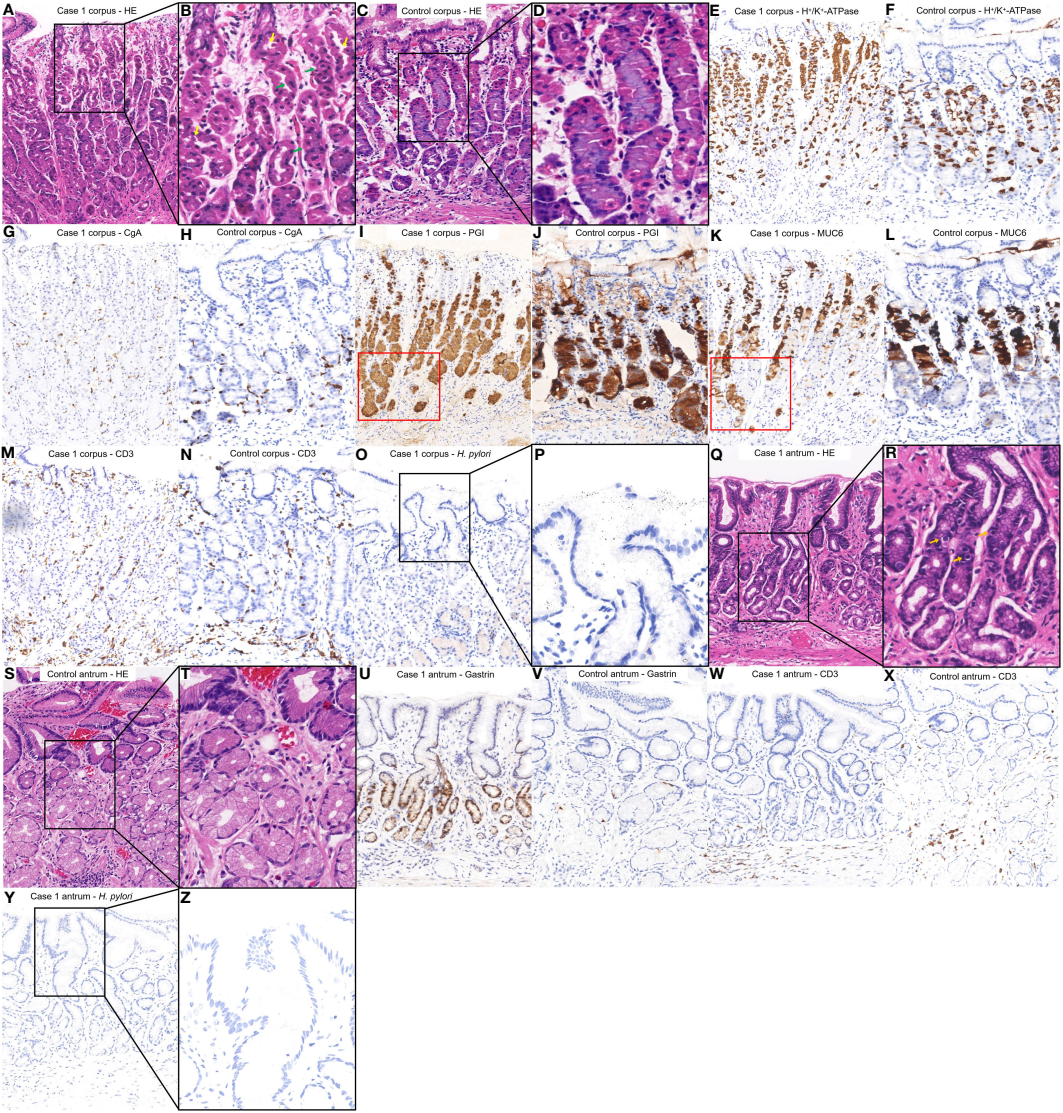
Histopathological findings from different regions of the patient’s stomach in Case 1 and a healthy control case. **(A, B)** Tissue obtained from the corpus of the patient in Case 1 revealed the arrangement of the parietal cells exhibiting irregularities, characterized by pseudohypertrophy (green arrows) and protrusions into the lumen (yellow arrows). **(C, D)** Normal corpus. **(E, G, I, K, M, O, P)** were comparisons of differential staining in the same region of **(A, F, H, J, L, N)** were comparisons of differential staining in the same region of **(C)**. **(E)** H+/K+ATPase staining revealed areas where the parietal cells remained. **(F)** Normal H^+^/K^+^ATPase staining. **(G)** Chromogranin A staining exhibited no significant proliferation of enterochromaffin-like cells. **(H)** Normal Chromogranin A staining. **(I, K)** Pepsinogen I and MUC6 staining revealed that mucous neck cells appeared at the base of corpus glands (red square). **(J)** Normal Pepsinogen I staining. **(L)** Normal MUC6 staining. **(M)** CD3 staining revealed a small amount of lymphocytic infiltration. **(N)** Normal CD3 staining. **(O, P)** *H. pylori* staining revealed absence of *H. pylori.* **(Q, R)** The tissue obtained from the antrum of the patient in Case 1 revealed an absence of inflammation or atrophy but G cell hyperplasia (Orange arrows). **(S, T)** Normal antrum. **(U, W, Y, Z)** were a comparison of differential staining in the same region of **(Q, V, X)** were a comparison of differential staining in the same region of **(S)**. **(U)** Gastrin staining indicated the proliferation of G cells hyperplasia. **(V)** Normal Gastrin staining. **(W)** CD3 staining revealed a lack of significant lymphocytic infiltration. **(X)** Normal CD3 staining. **(Y**, **Z)** *H. pylori* staining revealed absence of *H. pylori*.

Endoscopic examination revealed a normal antral mucosa (**Figure 1A**). The greater curvature of the corpus exhibited an irregular distribution of folds with deep fissure-like structures and a localized yellowish-white color (**Figure 1B**). Yellowish-white cobblestone-like elevations were observed in the upper part of the lesser curvature of the corpus. Closer examination of the surface revealed an irregular distribution of localized CVs (**Figures 1C, D**). In addition, the lesser curvature of the corpus displayed localized whitish areas with elongated and widened CVs on the surface. Magnifying endoscopy with narrowband imaging did not reveal any significant mucosal swelling (**Figures 1E, F**).

(**Figures 2A–D**) shows the HE staining results of corpus; (**Figures 2E, F**) shows the H+/K+ ATPase staining results of corpus; (**Figures 2G, H**) shows the CgA staining results of corpus; (**Figures 2I, J**) shows the PGI staining results of corpus; (**Figures 2K, L**) shows the MUC6 staining results of corpus; (**Figures 2M, N**) shows the CD3 staining results of corpus; (**Figures 2O, P**) shows the H. pylori staining results of corpus; (**Figures 2Q–T**) shows the HE staining results of antrum; (**Figures 2U, V**) shows the Gastrin staining results of antrum; (**Figures 2W, X**) shows the CD3 staining results of antrum; (**Figures 2Y, Z**) shows the H. pylori staining results of antrum. Among them, histopathological examination of tissue obtained from the gastric corpus showed no significant atrophy of the gastric glandular cells; however, the arrangement of the parietal cells began to exhibit irregularities characterized by hyperplasia and protrusions into the lumen (**Figures 2A, B**). H^+^/K^+^ ATPase staining revealed no significant detachment of the parietal cells (**Figure 2E**). Chromogranin A staining revealed no significant proliferation of enterochromaffin-like cells (**Figure 2G**). Pepsinogen I and MUC6 staining showed that the mucous neck cells appeared at the base of corpus glands (**Figures 2I, K**). CD3 staining revealed a small amount of lymphocytic infiltration (**Figure 2M**). *H. pylori* staining did not reveal the presence of *H. pylori* (**Figures 2O, P**).

Histopathological examination of biopsy specimens from the antrum revealed no atrophy or inflammation but G cell hyperplasia (**Figures 2Q, R**). Gastrin staining indicated the proliferation of G cells (**Figure 2U**). CD3 staining revealed no significant lymphocytic infiltration (**Figure 2W**). *H. pylori* staining did not reveal the presence of *H. pylori* (**Figures 2Y, Z**).

Serological testing revealed a positive outcome for the anti-parietal cell antibody and negative results for anti-intrinsic factor antibody and *H. pylori* antibody. Additionally, the anti-thyroid peroxidase antibody level was significantly elevated at 90.12 IU/ml (normal range: 0–5.61 IU/ml). The other test results were within normal limits. The left column of **Supplementary Table S1** shows the patients’ laboratory data.

In summary, the patient was diagnosed with early-stage AIG through the following features: (i) Medical history: family history of autoimmune disease and no history of PPI administration and *H. pylori* infection. (ii) Serology: PCA positive. (iii) Histopathology: A major histopathologic change of pseudohypertrophy and protrusion of parietal cells into the lumen is present with G cell hyperplasia, lymphocytic infiltration and possibly pseudopyloric gland metaplasia.

## 3 Case 2

A 70-year-old man underwent upper gastrointestinal endoscopy in July 2023 due to complaints of “postprandial fullness.” Prior to this assessment, he had not been prescribed medications, such as proton pump inhibitors. A positive urea breath test indicated the presence of *H. pylori* infection. Surprisingly, 2 weeks later, the serum *H. pylori* antibody test yielded negative results. Importantly, the patient had never undergone eradication therapy for *H. pylori*. His medical history revealed the presence of thyroid nodules, with normal thyroid function and antibody levels. Notably, the patient’s brother had a history of thyroid cancer.

**Figure 3:**
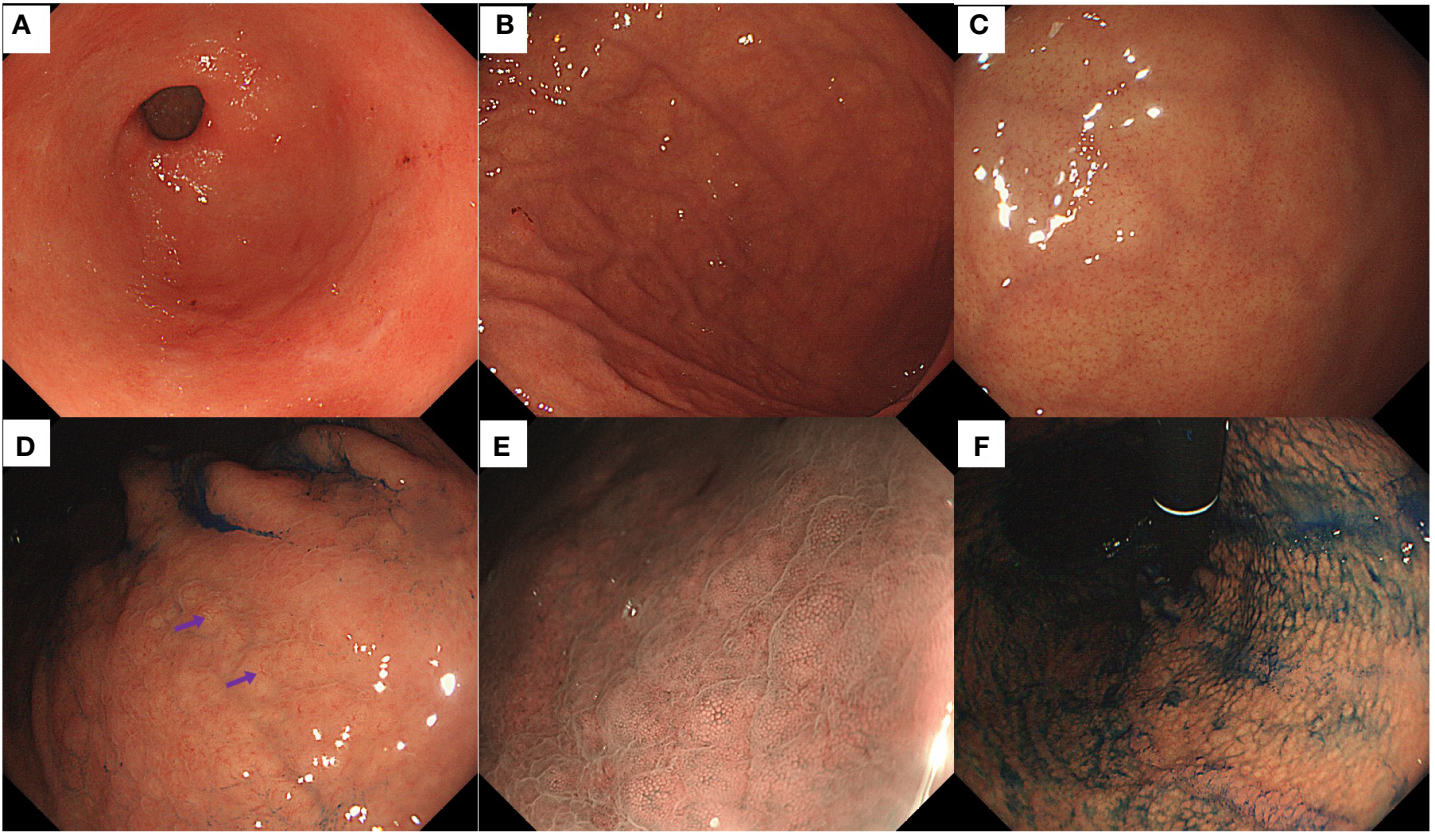
Endoscopic findings of the patient in Case 2. **(A)** Normal antrum. **(B)** Smooth mucosa with consistently thick folds were present in the greater curvature of the corpus. **(C)** Regular arrangement of CVs was visible on the mucosal surface of the fundus. **(D)** Some yellowish-white cobblestone-like elevations were present in the lesser curvature of the corpus, and an irregular distribution of CVs (purple arrows) on the surface was observed. **(E)** Magnifying endoscopy with narrow-band imaging revealed distinct CVs on the surface of the swollen mucosa. **(F)** The indigo carmine staining highlighted the gastric-area swelling.

**Figure 4:**
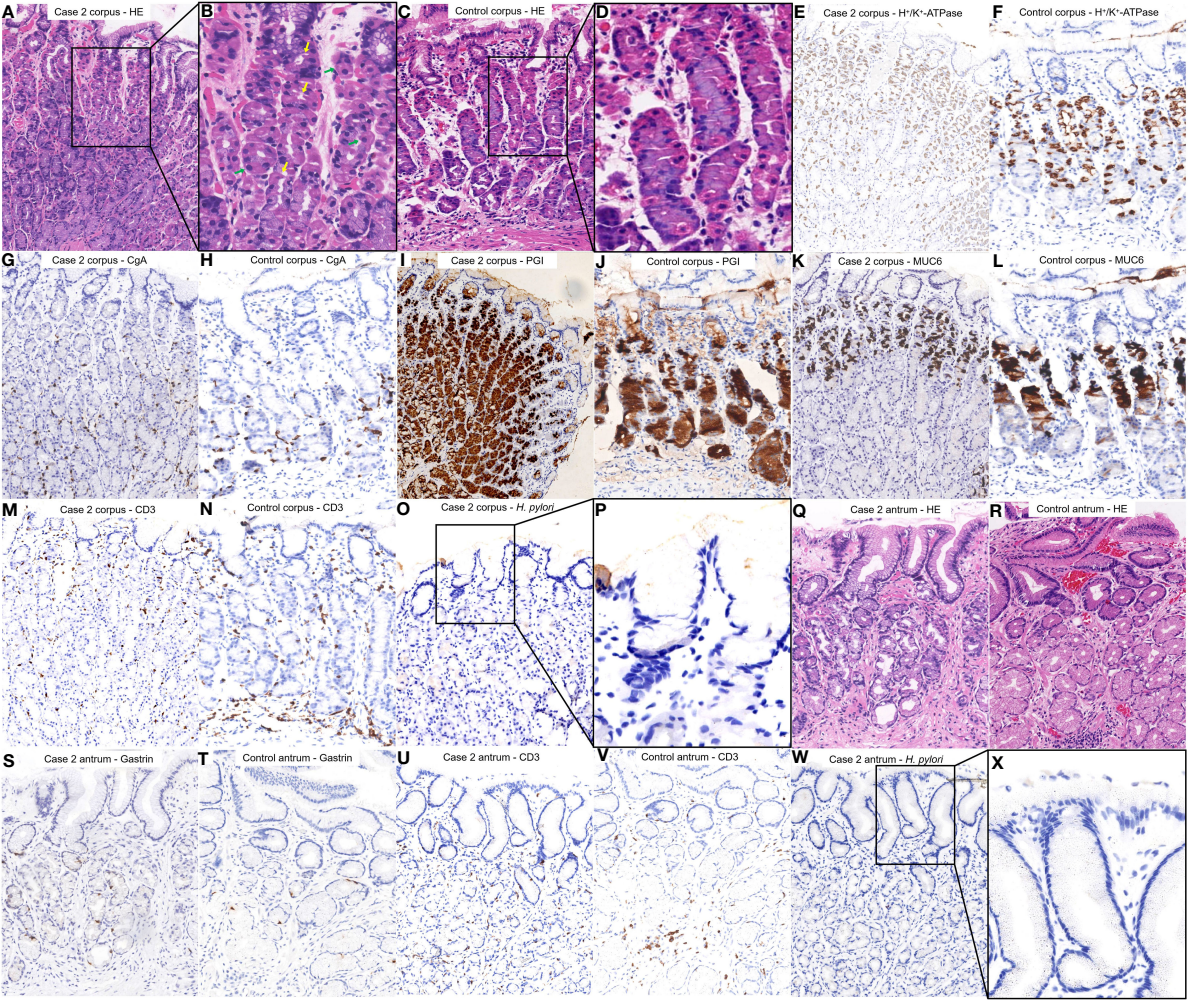
Histopathological findings from different regions of the patient’s stomach in Case 1 and a healthy control case. **(A, B)** Tissue was obtained from the corpus of the patient in Case 1. The parietal cells began to pseudohypertrophy (green arrows) and protrusion into the lumen (yellow arrows). **(C, D)** Normal corpus. **(E, G, I, K, M, O, P)** were comparisons of differential staining in the same region of **(A, F, H, J, L, N)** were comparisons of differential staining in the same region of **(C)**. **(E)** H+/K+ATPase staining revealed that the number of parietal cells was not significantly reduced. **(F)** Normal H^+^/K^+^ATPase staining. **(G)** Chromogranin A staining exhibited no significant proliferation of enterochromaffin-like cells. **(H)** Normal Chromogranin A staining. **(I)** Pepsinogen I staining revealed that chief cells were preserved. **(J)** Normal Pepsinogen I staining. **(K)** MUC6 staining revealed that mucous neck cells were preserved. **(L)** Normal MUC6 staining. **(M)** CD3 staining revealed no significant lymphocytic infiltration. **(N)** Normal CD3 staining. **(O**, **P)** *H. pylori* staining revealed absence of *H. pylori.* **(Q)** Tissue was obtained from the antrum of the patient in Case 1. No inflammation or atrophy was present. **(R)** Normal antrum. **(S**, **U**, **W**, **X)** were a comparison of differential staining in the same region of **(Q, T, V)** were a comparison of differential staining in the same region of **(R)**. **(S)** Gastrin staining did not demonstrate notable proliferation of G cells. **(T)** Normal Gastrin staining. **(U)** CD3 staining revealed no significant lymphocytic infiltration. **(V)** Normal CD3 staining. **(W, X)** *H. pylori* staining revealed absence of *H. pylori*.

Endoscopic examination revealed no inflammation or atrophy of the antral mucosa (**Figure 3A**). Smooth mucosa with consistently thick folds was observed in the greater curvature of the corpus (**Figure 3B**). The fundus mucosa displayed no signs of atrophy and exhibited a distinct and orderly arrangement of CVs (**Figure 3C**). Yellowish-white cobblestone-like elevations were noted on the lesser curvature of the corpus, indicating an irregular morphology and distribution of CVs upon closer examination (**Figure 3D**). Magnifying endoscopy with narrow-band imaging revealed distinct CVs on the surface of the swollen mucosa (**Figure 3E**). Indigo carmine staining revealed gastric area swelling (**Figure 3F**).

(**Figures 4A–D**) shows the HE staining results of corpus; (**Figures 4E, F**) shows the H+/K+ ATPase staining results of corpus; (**Figures 4G, H**) shows the CgA staining results of corpus; (**Figures 4I, J**) shows the PGI staining results of corpus; (**Figures 4K, L**) shows the MUC6 staining results of corpus; (**Figures 4M, N**) shows the CD3 staining results of corpus; (**Figures 4O, P**) shows the H. pylori staining results of corpus; (**Figures 4Q, R**) shows the HE staining results of antrum; (**Figures 4S, T**) shows the Gastrin staining results of antrum; (**Figures 4U, V**) shows the CD3 staining results of antrum; (**Figures 4W, X**) shows the H. pylori staining results of antrum. Among them, histopathological examination of the tissue obtained from the gastric corpus revealed pseudohypertrophy of the parietal cells protruding into the lumen (**Figures 4A, B**). Staining for H^+^/K^+^ ATPase (**Figure 4E**), Pepsinogen I (**Figure 4I**), and MUC6 (**Figure 4K**) revealed that the parietal cells were not significantly diminished, while chief cells and mucous neck cells were well preserved. Chromogranin A staining revealed no significant proliferation of enterochromaffin-like cells (**Figure 4G**). CD3 staining did not indicate notable lymphocytic infiltration (**Figure 4M**). *H. pylori* staining did not reveal the presence of *H. pylori* (**Figures 4O, P**).

Histopathological examination of the tissue collected from the antrum showed no significant signs of inflammation or atrophy (**Figure 4Q**). Gastrin staining did not show significant proliferation of G cells (**Figure 4S**). CD3 staining did not indicate notable lymphocytic infiltration (**Figure 4U**). *H. pylori* staining did not reveal the presence of *H. pylori* (**Figures 4W, X**).

Serology results indicated a positive anti-parietal cell antibody and negative findings for anti-intrinsic factors and *H. pylori* antibodies. Additionally, the patient’s hemoglobin level was measured at 128.00 g/L, which was slightly below the normal range of 130–175 g/L. The antithyroglobulin antibody level was recorded at 4.70 IU/ml, which was slightly elevated compared to the standard reference range of 0–4.11 IU/ml. Other laboratory test results were within normal ranges. The right column of **Supplementary Table S1** shows the patients’ laboratory data.

In summary, the patient was diagnosed with early-stage AIG through the following features: (i) Medical history: history of thyroid disease and no history of PPI administration and *H. pylori* infection. (ii) Serology: PCA positive. (iii) Histopathology: A major histopathologic change of pseudohypertrophy and protrusion of parietal cells into the lumen is present.

## 4 Discussion

Two patients suspected of having early-stage AIG on upper gastrointestinal endoscopy displayed the following characteristics: First, there was no atrophy of the gastric mucosa, either in the pyloric or fundic gland region. Second, the CVs in the fundic gland region exhibited irregular arrangements in localized regions that were either elongated, widened, or sparsely distributed. Third, yellowish-white cobblestone-like elevations were present in the fundic gland region, similar to the cobblestone-like changes induced by proton pump inhibitor usage. However, these elevations differed in color from the surrounding mucosa, and irregular CVs were visible on the surfaces. It is crucial to emphasize that in Case 1, the patient’s gastric mucosa did not display significant swelling, whereas in Case 2, mild swelling was observed along the lesser curvature of the gastric corpus.

Both of our patients fulfilled the diagnosis of early-stage AIG. The guideline defines the diagnosis of AIG as a positive gastric autoantibody (PCA and/or IFA), adding to endoscopic manifestations and/or histopathologic manifestations fulfilling the features of AIG (12). In addition, it characterizes the histopathology of early-stage AIG as: (i) The normal two-layered structure of fundic glands, i.e., the parietal cell/mucous neck cell layer and the chief cell layer are obscured, although the parietal and mucous neck cell layer is preserved. (ii) Many parietal cells remain, although swelling (swelling = pseudohypertrophy), intraluminal protrusion, and shedding are observed. (iii) Chief cells often transform to pyloric gland cells/mucous neck cells (pseudopyloric gland cells). (iv) Hyperplasia of ECL is either absent or mild, and mild-to-moderate lymphocytic/plasma cell infiltration is observed between the gastric glands. (v) Gastrin (G) cells in the pyloric gland mucosa also show mild hyperplasia in the early stage. However, it should be noted that these changes in parietal cells are key findings for early stage. Moreover, lymphocytic infiltration/plasma cell infiltration in the stroma between the oxyntic glands and hyperplasia of G cells in the pyloric glands is also used as a diagnostic aid. In our study, two cases were characterized by the following features: (i) Medical history: history of autoimmune disease or family history and no history of PPI administration and *H. pylori* infection. (ii) Serology: positivity for PCA. (iii) Histopathology: key histopathologic changes such as pseudohypertrophy and protrusion of parietal cells into the lumen, possibly along with hyperplasia of G cells, lymphocytic infiltration and potentially pseudopyloric gland metaplasia. In summary, we believe that these two cases are consistent with the serologic features and major histopathologic features of early-stage AIG, supported by a history of autoimmune disease or family history, and excluding the interference of PPI and *H. pylori*. Therefore, we believe that these two cases can be diagnosed as early-stage AIG.

Swelling of the gastric mucosa is an important indication of inflammation and a key point that draws the attention of the endoscopist (15). According to a prior study by Ayaki M et al. (16), a mosaic pattern with mild mucosal swelling confined to the corpus could indicate early-stage AIG. Nevertheless, our study indicated that certain cases of early-stage AIG might present with minimal gastric mucosal swelling or even in the absence of any observable swelling. Recently, Kotera et al. (17) documented a case of early-stage AIG that presented with a normal endoscopic appearance. Their findings indicated that during the early phases of AIG, a limited number of lymphocytes may not lead to observable endoscopic alterations in the gastric mucosa because of the infiltration of these lymphocytes into the deeper layers. In this study, the gastric mucosa of the patient in Case 1 exhibited no significant swelling, and the pathology revealed a small amount of lymphocytic infiltration, which was consistent with the results reported by Kotera et al. (17). Conversely, the patient in Case 2 displayed mild mucosal swelling in the lesser curvature of the gastric body, but the pathology did not reveal lymphocytic infiltration. Considering that the swollen mucosa was primarily situated in a region displaying a yellowish-white, cobblestone-like elevation, we postulated that the swelling was associated with an elevation caused by parietal cell degeneration. Consequently, while gastric mucosal swelling serves as a supplementary diagnostic indicator for early-stage AIG, it is not essential for diagnosing AIG at this stage.

Irregular CVs observed on the surface of the gastric mucosa may represent a characteristic feature of early-stage AIG. CVs represent blood vessels that begin at the level of the gastric foveolar layer and descend to merge with the submucosal plexus to form a complex and tree-like three-dimensional structure. Their morphology and arrangement are intricately linked to the presence and organization of gastric foveoli and body glands (18). Kishino et al. (19) documented a case of early-stage AIG. Well-preserved microvascular and microsurface patterns were observed in the gastric mucosa within the erythematous regions of the fundic glands. Their findings indicated that this preservation could be attributed to a more profound inflammation in the lamina propria mucosa than in the subepithelial region. When *H. pylori* infection is absent, normal gastric mucosa features a distinctive regular arrangement of CVs (20–22). When *H. pylori* infects the gastric mucosa, superficial inflammation causes elongation of the gastric pits, thus rendering these venules invisible on the mucosal surface (23). In early-stage AIG, lymphocytic infiltration originates from deeper layers, allowing for the possible observation of CVs if the gastric pits remain unaffected. However, unlike the regular arrangement of CVs, their morphology and arrangement may be altered during this phase, presenting irregularities, such as elongation, broadening, or sparse distribution. This phenomenon may be attributed to pseudohypertrophy and luminal protrusion of the parietal cells, resulting in a subtle distortion of the glands. In this report, a deviation from the typical arrangement of CVs observed in normal gastric mucosa was noted on the surface of the gastric mucosa in two patients with early-stage AIG. Moreover, biopsies demonstrated pseudohypertrophy of parietal cells and their protrusion into the lumen, providing substantial evidence to support the aforementioned concept. Based on these observations, we hypothesized that irregular patterns of CVs on the gastric mucosal surface were associated with early-stage AIG.

Yellowish-white cobblestone-like elevations may be new clues for recognizing early-stage AIG. Yellowish-white cobblestone-like elevations were observed in the lesser curvature of the gastric body in both patients with early-stage AIG. These are similar to the cobblestone-like changes reported previously. It is crucial to differentiate these features from the classic cobblestone-like alterations typically associated with prolonged proton pump inhibitor use in *H. pylori*-negative individuals and patients with middle-stage AIG (24). For example, cobblestone-like changes formed by the long-term administration of proton pump inhibitors are caused by parietal cell protrusion and demonstrate similar coloring to the surrounding mucosa (12). However, both cases of early-stage AIG showed coloration inconsistent with that of the surrounding mucosa, displaying yellowish-white cobblestone-like elevations. In addition, the cobblestone-like changes observed in middle-stage AIG result from the remnant oxyntic mucosa within the atrophic regions (25). Unlike these two cases of early-stage AIG, the yellow-white cobblestone-like elevations were surrounded by non-atrophic mucosa. Furthermore, a normal white zone was observed on the yellowish-white cobblestone surfaces. Although irregular CVs were observed on the surface, tumor-related lesions were not considered (26). In line with the pathology, we propose that the yellow-white cobblestone-like elevations in the region of the gastric fundus glands were caused by pseudohypertrophy and protrusion of parietal cells into the lumen. Therefore, yellow-white cobblestone-like elevations are different from the previously recognized cobblestone-like changes, which are expected to be a new clue for diagnosing early-stage AIG.

The histological changes observed in the two patients supported the diagnosis of early-stage AIG. There was a lack of significant lymphocytic infiltration in Case 2, which diverged from previous reports on early-stage AIG. Terao et al. (27) suggested that the identification of early-stage AIG should focus on the slight degeneration of the parietal cells and pseudopyloric gland metaplasia of the chief cells. Our histopathology revealed pseudohypertrophy of the parietal cells and pseudo-pyloric gland metaplasia, supporting the findings of Terao et al. Therefore, whether both lymphocytic infiltration and parietal cell degeneration are necessary, and the sequence of their appearance needs to be further clarified. Moreover, the overlapping staining of Pepsinogen I and MUC6 at the low base in Case 1 can be used as a clue for the diagnosis of early-stage AIG. Pepsinogen I and MUC6 staining usually have some overlap in the high base/low neck region of corpus glands, which is related to the distribution of mucous neck cells near the neck of the glands. However, for the overlapping staining at the low base of the gland, we thought it might appear in two cases: (i) The overlapping area had both chief cells and mucus neck cells, suggesting that the mucus neck cells had appeared at the base of the gastric gland. (ii) Only mucus neck cells were present in the overlapping area, suggesting that the chief cells had been replaced by pseudopyloric gland metaplasia. According to the latest diagnostic criteria for AIG (12), a significant increase in mucous neck cells at the base of the gastric glands, distribution of mucus neck cells throughout the gastric gland, and pseudopyloric gland metaplasia are all pathological manifestations of early-stage AIG. Therefore, the overlapping staining of Pepsinogen I and MUC6 at low base can serve as a diagnostic clue for early-stage AIG.

Serological tests play a crucial role in the diagnosis of early-stage AIG (28). The specificity of anti-parietal cell antibodies is somewhat limited (29, 30); however, more attention should be paid to the diagnosis of early-stage AIG. Indeed, since there are few diagnostic clues for early-stage AIG, positivity for PCA is an extremely important clue when an early autoimmune response is initiated and atrophy has not yet occurred. Serological tests in both patients suggested anti-parietal cell antibody positivity, supporting the diagnosis of early-stage AIG. Furthermore, the evaluation of gastrin, pepsinogen, vitamin B12, iron, and autoimmune antibodies, and family medical history provided additional supportive evidence for the diagnosis of early-stage AIG to some extent. However, some patients with early-stage AIG may not develop hypergastrinemia and pernicious anemia due to mild parietal cell destruction. Gastrin and vitamin B12 levels in the two reported patients were normal, suggesting that their parietal cells might not yet be significantly disrupted. This speculation was corroborated by our histopathology, which observed only major changes in the pseudohypertrophy and protrusion into the lumen of parietal cells without reporting significant destruction of parietal cells. In addition, although we detected positive PCA, it takes time from PCA production to noticeable parietal cell destruction and intrinsic factor deficiency. Close follow-up of these patients in the coming years is essential to track the changes in their condition. Through this process, we can understand the progression of early-stage AIG and validate the current diagnosis.

## 5 Conclusion

Based on the two cases reported in this study and related literature, we summarized some endoscopic features of early-stage AIG: (1) Yellowish-white cobblestone-like elevations in the region of the fundic glands may be a new clue for the diagnosis of early-stage AIG. (2) Irregular CVs observed on the surface of the gastric mucosa in the fundic gland region may be a characteristic change in early-stage AIG. (3) Swelling of the gastric mucosa within the fundic gland region may also indicate early-stage AIG. Moreover, when considering an endoscopic diagnosis of early-stage AIG, it is imperative to perform further histological and serological examinations. Through these case reports, we emphasize the importance of endoscopic features of early-stage AIG and advocate for further attention and research focused on this disease.

## Data availability statement

The original contributions presented in the study are included in the article/**Supplementary Material**. Further inquiries can be directed to the corresponding author.

## Ethics statement

The studies involving humans were approved by Ethics Committee of The First Hospital of Hunan University of Chinese Medicine. The studies were conducted in accordance with the local legislation and institutional requirements. The participants provided their written informed consent to participate in this study. Written informed consent was obtained from the individual(s) for the publication of any potentially identifiable images or data included in this article.

## Author contributions

YY: Conceptualization, Supervision, Writing – original draft. XS: Data curation, Methodology, Writing – original draft. RY: Formal analysis, Writing – review & editing. YW: Data curation, Writing – original draft. EX: Data curation, Writing – original draft. CT: Conceptualization, Supervision, Writing – review & editing.
