# Supplementary material for: Unveiling early stage autoimmune gastritis: novel endoscopic insights from two case reports
Source: Front Immunol. 2024 Jun 17;15:1416292. doi: 10.3389/fimmu.2024.1416292 (PMC11215040; doi:10.3389/fimmu.2024.1416292)
Supplement: Supplementary file 1 [file Table_1.docx]

**Supplementary Table S1** Laboratory findings of Case 1 and Case 2.

| Indicators | Case 1 | Case 2 |
| --- | --- | --- |
| Anti‐parietal cell Ab. (-) | (+) | (+) |
| Anti‐intrinsic factor Ab. (-) | (-) | (-) |
| Anti‐Helicobacter pylori IgG Ab. (-) | (-) | (-) |
| Gastrin (1.5~7.5 pmol/L) | 3.34 | 1.96 |
| Pepsinogen I (30~200 ng/mL) | 49.28 | 85.29 |
| Pepsinogen II (0~15 ng/mL) | 7.00 | 13.21 |
| Pepsinogen I/II (>=3~) | 7.04 | 6.46 |
| Vit B12 (133~675 pmol/L) | 235.71 | 423.20 |
| Fe (11~30 umol/L) | / | 35.70 |
| RBC (4.3~5.8 *10∧12/L) | 4.64 | 3.83 |
| Hb (130~175 g/L) | 141.00 | 128.00 |
| MCV (82~100 fL) | 94.40 | 100.20 |
| TSH (0.35~4.94 u IU/ml) | 1.5230 | 1.9569 |
| FT4 (0.70~1.48 ng/dl) | 1.01 | 0.78 |
| Anti‐TPO Ab. (0~5.61 IU/ml) | 90.12 | 3.47 |
| Antithyroglibulin Ab. (0~4.11 IU/ml) | / | 4.70 |
| Anti-nuclear Ab. (-) | (-) | (-) |
| Anti‐ds‐DNA Ab. (-) | (-) | (-) |
| Anti‐SS‐A/Ro Ab. (-) | (-) | (-) |
| Anti‐SS‐B/La Ab. (-) | (-) | (-) |
| Anti‐RNP Ab. (-) | (-) | (-) |

Ab, antibody; dsDNA, double-stranded deoxyribonucleic acid; Fe, iron; FT4, free thyroxine; Hb, hemoglobin; MCV, mean corpuscular volume; RBC, red blood cells; RNP, ribonucleoprotein; SS, Sjögren’s syndrome; TPO, thyroid peroxidase; TSH, thyroid-stimulating hormone; Vit, vitamin.
